# Exploring Gut Microbiota Imbalance in Irritable Bowel Syndrome: Potential Therapeutic Effects of Probiotics and Their Metabolites

**DOI:** 10.3390/nu17010155

**Published:** 2024-12-31

**Authors:** María José García Mansilla, María Jesús Rodríguez Sojo, Andrea Roxana Lista, Ciskey Vanessa Ayala Mosqueda, Antonio Jesús Ruiz Malagón, Julio Gálvez, Alba Rodríguez Nogales, María José Rodríguez Sánchez

**Affiliations:** 1 Department of Pharmacology, Centro de investigación Biomédica (CIBM), University of Granada, 18071 Granada, Spain; garciamansillamariajose@gmail.com (M.J.G.M.); mariajesus.rodriguez.sojo@gmail.com (M.J.R.S.); jgalvez@ugr.es (J.G.); albarn@ugr.es (A.R.N.); mjrs2188@gmail.com (M.J.R.S.); 2 Instituto de Investigación Biosanitaria de Granada (ibs.GRANADA), 18012 Granada, Spain; andreea99.lista@gmail.com (A.R.L.); ciskeyay@correo.ugr.es (C.V.A.M.); 3 Instituto de Investigación Biomédica de Málaga y Plataforma en Nanomedicina-IBIMA Plataforma BIONAND, 29590 Málaga, Spain; 4 CIBER de Enfermedades Hepáticas y Digestivas (CIBER-EHD), Instituto de Salud Carlos III, 28029 Madrid, Spain

**Keywords:** Irritable bowel syndrome, gastrointestinal, treatment, probiotics, metabolites, symptoms

## Abstract

Irritable bowel syndrome is a common functional gastrointestinal disorder characterized by recurrent abdominal discomfort, bloating, cramping, flatulence, and changes in bowel movements. The pathophysiology of IBS involves a complex interaction between motor, sensory, microbiological, immunological, and psychological factors. Diversity, stability, and metabolic activity of the gut microbiota are frequently altered in IBS, thus leading to a situation of gut dysbiosis. Therefore, the use of probiotics and probiotic-derived metabolites may be helpful in balancing the gut microbiota and alleviating irritable bowel syndrome symptoms. This review aimed to report and consolidate recent progress in understanding the role of gut dysbiosis in the pathophysiology of IBS, as well as the current studies that have focused on the use of probiotics and their metabolites, providing a foundation for their potential beneficial effects as a complementary and alternative therapeutic strategy for this condition due to the current absence of effective and safe treatments.

## 1. Introduction

Irritable bowel syndrome (IBS) is a functional gastrointestinal (GI) disorder characterized by recurrent abdominal pain associated with abnormal stool form or frequency [1]. At present, IBS is classified according to the predominant clinical symptoms. The proposed subtypes are predominant constipation (IBS-C), predominant diarrhea (IBS-D), mixed bowel habits (IBS-M), and unclassified IBS [2]. Moreover, the presence of post-infection IBS (PI-IBS) has been confirmed by epidemiological research carried out in various geographical and clinical environments [2]. The global prevalence has been estimated to be up to 10% [3]. Being the patients usually diagnosed at a young age (20–30 years) and more prevalent in women [4]. Of note, although this chronic disorder does not increase mortality risk, it impairs the quality of life, and it has been associated with a substantial economic burden on patients and healthcare systems [5].

Unfortunately, and although the prevalence is high, its etiology is not fully understood. In this sense, there is a growing body of research involving both physiological and psychological factors responsible for IBS development and its associated symptoms [6].

The pathophysiology of IBS is complex and multifactorial; hence, it remains poorly understood. Several factors have been proposed as elicitors to IBS, including genetic pre-disposition and different environmental components such as diet, in association with altered gut-brain communication, visceral hypersensitivity, gut motility issues, innate immunity dysfunction, food intolerances, low-grade gut mucosal inflammation, psychosocial stressors, abnormalities in serotonin metabolism, alterations in brain function, and altered gut microbiome, among others [7,8,9,10]. However, the specific triggers or exacerbators of IBS symptoms can vary among individuals, making it challenging to pinpoint a single cause.

A great deal of research has documented the role of innate immune dysfunction and its impact on both systemic and mucosal low-grade inflammation. In patients with IBS, immune system activation in the colonic mucosa has been observed, accompanied by immune cell infiltration and the release of pro-inflammatory cytokines, including IL-6, TNF-α, IL-17, and IL-1β, among others [9,11,12]. Although the underlying cause of this altered immune response remains unclear, studies involving patients with IBS have shown increased intestinal permeability, suggesting a compromised mucosal epithelial barrier that could disrupt gut immune homeostasis, potentially leading to gut inflammation and atypical immune responses [13].

**Figure 1:**
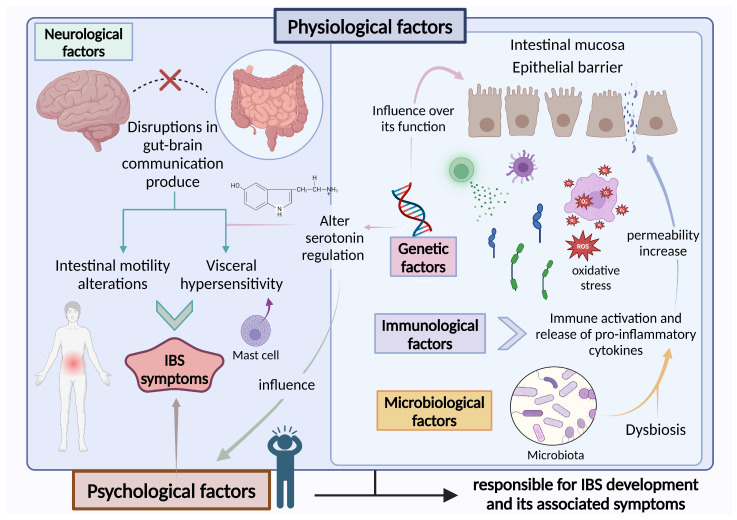
Pathophysiology of the IBS. The pathophysiology of IBS involves the interaction of psychological, genetic, microbiological, neurological, and immunological factors, which contribute to symptoms, dysbiosis, motility alterations, and immune dysregulation. IBS, Irritable Bowel Syndrome [9,10,13,14,15,17,18,19,20,25,26,28,29,30,31,32,38,39].

On the other hand, altered food-derived or bacterial products due to dysbiosis, or an impaired epithelial response, have been shown to encourage a pro-inflammatory dendritic cell phenotype, difficulting the induction of tolerogenic or regulatory mechanisms and favoring a type 2 (pro-inflammatory) immune response instead. Consequently, food antigen-specific IgE might trigger mast cell activation and visceral nociceptor sensitization. Alternatively, mast cell activation can be directly induced by bacterial or food-derived products, as well as through neurogenic inflammation and psychological stress [14]. Mast cell mediators, such as histamine and tryptase, have been shown to be released from colonic biopsies of patients with IBS and degranulated mast cells situated near nerves in the colonic mucosa. These mediators are associated with the severity and frequency of abdominal pain, reinforcing the idea that the role of mast cells is involved in visceral hypersensitivity (Figure 1) [15].

The influence of genetic factors on the evolution of IBS is still unclear because of relatively small study groups and a lack of significant structural anomalies. The role of both common and rare gene variants in IBS susceptibility is largely unidentified [16]. Recent research has identified polymorphisms in genes related to IBS pathogenesis, including genes responsible for epithelial barrier role, immune regulation [17], serotonin signaling [18,19], cannabinoid receptors [20], or BAs synthesis [21]. On the other hand, GWAS studies demonstrated GRID2IP (glutamate receptor, ionotropic delta 2 interacting protein), KDELR2 (endoplasmic reticulum protein retention receptor 2), and TNFSF15d (Tumor Necrosis Factor Superfamily) to be involved in IBS development [22,23]. Furthermore, epigenetic factors such as DNA methylation may play a role in IBS manifestation (Figure 1) [24].

Recent research combined with clinical observations underscores a vital function of the brain-gut axis in the development and persistence of IBS symptoms. Regardless of the main symptom triggers, the brain is ultimately accountable for molding and producing the conscious perception of abdominal pain, discomfort, and anxiety based on sensory information from the gut [25]. Stressful and traumatic events increase the likelihood of developing IBS, while psychosocial stressors significantly influence the initial onset, symptom flare-ups, and noticed severity of symptoms (Figure 1) [26,27].

Regarding the role of the microbiome in IBS development, many studies have demonstrated that diversity, stability, and metabolic function of the gut microbiota are often modified in individuals with IBS, resulting in a condition of gut dysbiosis (Figure 1) [28,29,30,31,32]. The human gut microbiota consists of a complex community constituted by more than 1500 species of microorganisms, including bacteria, viruses, and eukaryotes [33]. The gut microbiota plays a significant role in maintaining normal gut physiology and health, including supporting protection from pathogens [34], participating in digestion and metabolism [35], controlling epithelial cell differentiation and proliferation [36], or influencing brain–gut communication [37], among others. Although only about 30% of the bacterial species have been identified and described thus far, the GI tract predominantly includes *Bacillota* (64%), *Bacteroidota* (23%), *Pseudomonadota* (8%), and *Actinobacteriodota* (3%) [16]. Disruption of the gut microbiota may result in a condition known as dysbiosis and can happen due to the loss or excessive growth of a specific organism, a decrease in microbial diversity, or genetic mutations [38]. Recent findings indicate that gut dysbiosis may play an important function in the development and progress of IBS. The commensal organisms that normally inhabit the gut influence signaling molecules and metabolites essential for maintaining gut homeostasis and mucosal immune system formation [39]. Even minor changes in the gut microbiome can result in inflammatory responses that induce oxidative stress, heighten intestinal permeability, and may involve bacterial translocation across the mucosal layer [39]. Notable differences have been observed in the composition of the gut microbiome in patients with IBS. While researchers have recently pinpointed a signature gut microbiome potentially linked to severe IBS [27], characterization of the IBS intestinal microbiome remains inconsistent, and no definitive signature has gained acceptance [40]. An original investigation involving patients with IBS and matched controls without IBS identified an abundance of *Ruminococcus gnavus* and *Lachnospiraceae* and reduced levels of *Barnesiella intestinihominis* and Coprococcus catus [41]. A meta-analysis encompassing 13 studies found declines in *Bifidobacterium*, *Lactobacillus*, and *Faecalibacterium prausnitzii* among patients [42]. Another meta-analysis involving 777 patients and 16 studies showed elevated levels of *Bacillota* and diminished *Bacteroidota* (with an increased *Bacillota* ratio) at the phylum level. They also noted several alterations at lower taxonomic levels, including higher concentrations of *Clostridia* and *Clostridiales* and reduced concentrations of *Bacteroidia* and *Bacteroidales* [43]. Likewise, a meta-analysis involving 1340 participants from 23 studies found decreased levels of *Lactobacillus* and *Bifidobacterium*, alongside higher levels of *Enterobacter* and *Escherichia coli*, in the micro-biome analysis of individuals with IBS compared to controls. These researchers did not observe any differences in fecal *Bacteroides* or *Enterococcus* levels [44]. While there is evidence indicating that the microbiome varies between patients and controls, most studies have struggled to identify significant differences among IBS subtypes [41,43,45,46]. It is crucial to recognize that an inability to detect significant variations between microbiome phenotypes in individuals with IBS may, in part, stem from a lack of consistent methodologies. Whole-shotgun metagenomics is currently the standard technology employed for analyzing gut microbial compositions; however, this method relies on bioinformatic pipelines to interpret the data, which carry their own limitations [46,47,48]. Additionally, it is important to take into account that only with taxonomic composition may the differences in functional phenotypes between individuals be defined, highlighting the necessity of utilizing metatranscriptomics, metagenomics, and metabolomics when exploring these functional distinctions. A recent study involving a cohort of 495 subjects, including 318 patients with IBS and 177 controls, indicated that variations occurred not only in the microbiome composition of patients with IBS but also in metabolites and transcripts related to fructooligosaccharide utilization. This study also demonstrated metatranscriptomic and metabolomic variations between IBS-D and IBS-C subtypes [48].

Researchers have also determined a role for the gut virome in IBS, noting significantly lower alpha diversity and different beta diversity in patients with IBS, with the most prevalent viral clusters recognized as belonging to the *Siphoviridae*, *Myoviridae*, and *Podoviridae* families [46]. It is also significant to mention that there may be a correlation between the gut microbiome and psychological conditions. One study revealed that the fecal microbiota of IBS-D patients was similar to that of individuals with depression. For this purpose, samples from 100 subjects (40 with IBS-D, 15 with depression, 25 with comorbidities of IBS and depression, and 20 controls) showed reduced overall diversity and increased abundance of *Bacteroides*, *Prevotella*, or nondominant microbiota [49]. Moreover, it has been discovered a significant correlation between the gut microbiome in patients with IBS and psychological distress, anxiety, and depression [50]. More data are required to comprehend the significance of this correlation in the development of IBS and/or psychiatric conditions. Given the extensive variability in microbiome data related to IBS, additional studies incorporating metabolomic, metatranscriptomic, and metagenomic sequencing are essential for better characterizing the signature gut microbiome and understanding its role in various diseases.

Experimental evidence obtained from germ-free mice colonized with human microbiota has supported a role of the gut bacteria in IBS pathogenesis, including its psychological comorbidity [51]. Of note, most of the recent research connecting IBS and gut microbiota has primarily focused on alterations in the composition and function of intestinal microbiota. Nevertheless, with advancements in molecular biology tools and methodologies, it has been proposed that the microbial byproducts can play an important function in the interactions between gut microbiota and the intestinal epithelial mucosal immune system in the gut, which significantly influence IBS [52]. Confirming this, it has been reported that gut dysbiosis in subjects with IBS was associated with a shift in their metabolic profile, including variations in short-chain fatty acids (SCFAs), tryptophan (Trp) metabolites, and bile acids (BAs), which could be linked to the pathogenesis of IBS [53].

In this context, the development of microbiome-metabolome modulator agents emerges as a potential therapeutic strategy. In fact, probiotics have been long considered as promising agents in the prevention and management of IBS, based on their well-known beneficial effects by regulating the intestinal micro-ecosystem, enhancing the integrity of the epithelial barrier, and reducing immune and inflammatory responses [54,55]. However, there are limited clinical studies on the impact of probiotics and/or their metabolites on IBS. It is relevant to note that some findings have revealed some concerns with the administration of probiotics in these conditions because their effectiveness is influenced by different factors such as species and/or strain selected; also, the dosage and treatment duration may also vary based on clinical indications.

In this review, the role of gut dysbiosis in the pathophysiology of IBS will be evaluated, thus establishing the basis for the potential beneficial effects of probiotics and their metabolites as a complementary and alternative therapeutic approach in this condition, given the lack of effective and safe treatments at present.

## 2. Role of Probiotics in IBS

**Figure 2:**
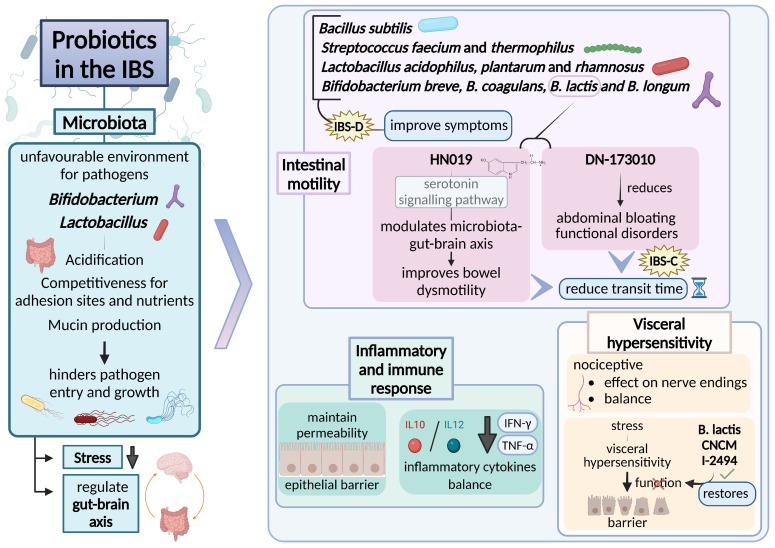
Beneficial effects of probiotics in managing IBS through modulation of the gut microbiota. Probiotics improve intestinal motility, balance inflammatory responses, reduce visceral hypersensitivity, and regulate stress and microbiota-gut-brain axis. IBS, irritable bowel syndrome; IBS-C, IBS-predominant constipation; IBS-D, predominant diarrhea [56,58,59,61,62,63,66,67,71,72,73,74,75,76,83,84,85,86,87,88,89,90].

Experimental and clinical research over recent years has identified several mechanisms by which probiotics beneficially impact the pathophysiological processes involved in IBS (Figure 2) [56]. Table 1 provides an overview of the bacterial strains frequently used in probiotic formulations. Probiotics play an essential role in relieving IBS symptoms like gas, abdominal discomfort, and bloating [57]. They influence microbiota composition, enhancing intestinal motility, visceral sensitivity, immune function, and metabolic activity, thereby contributing positively to microbiota-gut-brain axis regulation and related psychiatric conditions. The key benefits reported in the literature are summarized in Table 1.

**Table 1:** Bacterial strains commonly utilized in probiotic formulations and their beneficial effects reported in the literature.

Beneficial Effect	Probiotic Strain	Studies
Restoring Microbiota Composition	*Bifidobacterium animalis subsp. lactis, BB-12*	[58]
	*Lactobacillus acidophilus* *Lactobacillus casei*	[59]
	*Lactobacillus paracasei D3-5, Lactobacillus rhamnosus D4-4, Lactobacillus plantarum D6-2, Lactobacillus rhamnosus D7-5 and Lactobacillus plantarum D13-4, Enterococcus raffinosus D24-1, Enterococcus INBio D24-2, Enterococcus avium D25-1, Enterococcus avium D25-2 and Enterococcus avium D26-1*	[60]
Enhancing Intestinal Motility	*Bifidobacterium lactis HN019*	[61,62]
	*Bifidobacterium lactis DN-173 010*	[63]
	*Bacillus subtilis* *Enterococcus faecium* *Enterococcus faecalis*	[64]
	*Lactobacillus rhamnosus GG* *Bifidobacterium animalis subsp. lactis BB-12*	[65]
	*Bifidobacterium animalis subsp. lactis, BB-12*	[58]
	*Bacillus subtilis* *Streptococcus faecium*	[66]
	*Lactobacillus coryniformis CECT5711* *Lactobacillus gasseri CECT5714*	[67]
	*Bacillus coagulans Unique IS2*	[68,69,70,71]
Visceral Hypersensitivity	*Bifidobacterium infantis 35624*	[71]
	*Bifidobacterium animalis subsp. lactis, Streptococcus thermophiles, Lactobacillus bulgaricus, and Lactococcus lactis subsp. lactis*	[72]
	*Bifidobacterium animalis subsp. lactis CNCM I-2494*	[73,74]
Modulation of Inflammatory and Immune Responses	*Lactococcus lactis*	[75]
	*Lactobacillus acidophilus NCFM™* *Lactobacillus salivarius Ls-33* *Bifidobacterium infantis 35624* *Escherichia coli Nissle 1917* *Saccharomyces boulardii* *VSL#3: Lactobacillus acidophilus, Lactobacillus plantarum, Lactobacillus casei, Lactobacillus delbrueckii subsp. bulgaricus, Bifidobacterium breve, Bifidobacterium longum, Bifidobacterium infantis and Streptococcus salivarius subsp. thermophilus*	[76]
Stress Response	*Lactobacillus rhamnosus R0011 and Lactobacillus helveticus R0052*	[77]
	*Bifidobacterium longum R0175 and Lactobacillus helveticus R0052*	[78,79]
	*Lactobacillus rhamnosus GG*	[80]
	*Bifidobacterium infantis 35624*	[76]
	*Bifidobacterium longum 35624*	[81]
	*VSL#3: Lactobacillus acidophilus, Lactobacillus plantarum, Lactobacillus casei, Lactobacillus delbrueckii subsp. bulgaricus, Bifidobacterium breve, Bifidobacterium longum, Bifidobacterium infantis, and Streptococcus salivarius subsp. thermophilus*	[82]

### 2.1. Impact of Probiotics in Restoring Microbiota Composition

In the absence of gut microbiota, the gut immune system stays underdeveloped, leading to fewer functional regulatory CD4+ CD25+ T cells and, as a consequence, a weakened capacity to defend against pathogenic organisms [91]. Furthermore, the equilibrium between pro-inflammatory IL-17-producing T cells and regulatory Forkhead box P3 (Foxp3+) T cells in the gut relies on signals from gut bacteria, and these signals are influenced by the composition of the intestinal microbiota (Figure 2) [56].

Probiotics can contribute to reestablishing the balance and composition of the gut microbiome and enhance the beneficial activities of gut microbial communities, leading to improvements or prevention of gut inflammation and other intestinal disease phenotypes [92]. Increasing levels of *Lactobacilli* and *Bifidobacteria* through probiotics intake have been shown to restore and stabilize an unfavorable intestinal environment for pathogenic microorganisms [58]. Specifically, *Lactobacilli*, through the production of lactic acid, contribute to the generation of an acidic environment that suppresses the growth of harmful bacteria. Additionally, it plays a role in eliminating pathogens by competing for adhesion sites and nutrients. This competition for nutrients hinders the growth of pathogenic microbes, particularly certain species of *Clostridium*, *Escherichia coli*, *Salmonella*, *Shigella*, and *Pseudomonas*. *Lactobacilli* and other probiotics have been demonstrated to enhance mucin release and control the synthesis of tight junction proteins, which helps block the entry and adhesion of toxins and pathogens (Figure 2) [59,83].

### 2.2. Effect of Probiotics in Enhancing Intestinal Motility

Many studies have evidenced optimized transit in patients with constipation. The administration of *Bifidobacterium lactis* HN019 and *Bifidobacterium lactis* DN-173 010 resulted in a shorter transit time in adult individuals suffering from chronic constipation [61,63]. Both in vitro and clinical studies have shown that *Bifidobacterium lactis* HN019 reduced intestinal transit time in functional constipation by modulating the gut-brain-microbiota axis, primarily through the serotonin signaling pathway. Therefore, *Bifidobacterium lactis* HN019 represents a probiotic capable of improving disorders related to intestinal dysmotility [61,62]. Fermented dairy products with *Bifidobacterium lactis* DN-173 010 reduced both abdominal bloating and transit time in a group of IBS-C patients [63]. Daily intake of *Bifidobacterium lactis* decreased the incidence of functional disorders in patients with abnormal transit and gas. The combination of *Bacillus subtilis* and *Streptococcus faecium* probiotics alleviated symptoms in patients with IBS without diarrhea [64]. A blend of probiotics including *Lactobacillus acidophilus*, *Lactobacillus plantarum*, *Lactobacillus rhamnosus*, *Bifidobacterium breve*, *Bifidobacterium lactis*, *Bifidobacterium longum*, and *Streptococcus thermophilus* improved symptoms in patients with IBS-D, yielding superior results without notable adverse effects [84].

A meta-analysis evaluating randomized controlled trials concluded that *Bifidobacterium lactis* intake reduced transit time in patients with chronic constipation [58,66,67,85,86]. A 2022 meta-analysis by Zhang et al. involving 43 clinical trials with 5.531 IBS patients suggested that *Bifidobacterium coagulans* is highly effective as a therapeutic agent for IBS-D patients, enhancing symptoms and quality of life [68]. This study identified *Bifidobacterium coagulans* as the most effective probiotic for improving abdominal pain and straining scores, maintaining substantial efficacy compared to various probiotic combinations. The authors emphasized the necessity for future research on this species, suggesting that developing specimens with enhanced biological functions through genetic engineering and creating probiotic combinations containing *Bifidobacterium coagulans* may be valuable research avenues (Figure 2) [68].

### 2.3. Probiotics in Visceral Hypersensitivity

Different studies involving animal models have demonstrated that probiotics promote a direct antinociceptive effect on sensitive nerve endings in the gut [71,87]. Other experiments support the theory that probiotics also modulate the balance between nociceptive and antinociceptive stimuli at the central nervous system level. In this sense, the administration of *Bifidobacterium Lactis* CNCM I-2494 was shown to be able to reduce stress-induced visceral hypersensitivity by restoring intestinal barrier function [73,74]. The administration of dairy products containing *Bifidobacterium animalis subsp. Lactis*, *Lactobacillus bulgaricus*, *Lactococcus lactis*, and *Streptococcus thermophilus* in healthy individuals was correlated with significant alterations in affective, viscerosensitive, and somatosensitive cortical processes, as observed in magnetic resonance imaging studies. This suggests a connection between probiotics and the activity of emotional processing centers (Figure 2) [72,88].

### 2.4. Probiotics and the Modulation of Inflammatory and Immune Responses

The association between IBS and the inflammatory and immune responses of the intestinal mucosa is indirectly indicated by the emergence of IBS symptoms following a bacterial or viral intestinal infection (IBS-PI). Recent research has shown that IBS is accompanied by alterations in both the local and systemic immune responses, both nonspecific and specific [93,94]. Increased permeability of the intestinal mucosa is recognized as a sign of local inflammation [89]. The non-specific local immune response is emphasized by the subepithelial buildup of mast cells, macrophages, and dendritic cells functioning as antigen-presenting cells. The non-specific local immune reaction is highlighted by the subepithelial accumulation of mast cells, macrophages, and dendritic cells acting as antigen-presenting cells. The non-specific systemic immune response manifests as elevated levels of certain cytokines: IL-1β, IL-6, IL-8, IL-12, and TNFα [95]. A reduction in the anti-inflammatory cytokine IL-10, which regulates the release of pro-inflammatory cytokines and antigen presentation, has also been noted; thus, IL-10 is proposed as a potent anti-inflammatory biological therapy for IBS [96]. Several laboratory findings and clinical studies illustrate that probiotics mitigate the inflammatory and immune responses in IBS via various mechanisms. Probiotics help maintain the common permeability of the epithelial barrier, correcting the imbalance between pro-inflammatory and anti-inflammatory cytokines (measured by the IL-10/IL-12 ratio) and lowering the local and systemic levels of several pro-inflammatory cytokines (TNF-α, IFN-γ) (Figure 2) [75,76].

### 2.5. Role of Probiotics in Stress Response

Extensive experimental and clinical evidence indicates bidirectional influences between the microbiota and the central nervous system. Dysbiosis can trigger alterations in the microbiota-gut-brain axis, while probiotics may assist in normalizing this interaction [90]. Several studies have emphasized the protective effects of probiotics against anxiety and depressive states induced by mental stress. Certain probiotics (strains of *Lactobacillus rhamnosus* and *Lactobacillus helveticus*) normalized the exaggerated response of the hypothalamic-pituitary-adrenal axis in IBS [77,90]. *Lactobacillus rhamnosus* reduced stress-induced corticosterone release by modulating GABA receptors involved in anxiety, thus decreasing the frequency and severity of abdominal pain episodes in patients with IBS (Figure 2) [78,79,80,97].

A strain of *Bifidobacterium longum* showed helpful effects in a recent study by Sabate et al., which determined that thirty days of *Bifidobacterium longum* 35624 treatment diminished disease intensity and improved the quality of life for patients with IBS, especially those with intense symptoms [76]. The stress-reducing effects induced by *Bifidobacterium* are likely linked to Trp metabolism, as increased levels of Trp were noted following probiotic administration [81,98]. A mixture of eight probiotic species (*Bifidobacterium longum*, *Bifidobacterium breve*, *Bifidobacterium infantis*, *Lactobacillus casei*, *Lactobacillus acidophilus*, *Lactobacillus plantarum*, *Lactobacillus delbrueckii subsp. Bulgaricus*, and *Streptococcus salivarius*) resulted in an increase in brain-derived neurotrophic factor (BDNF) levels. Dysfunctions in the epigenetic control, transport, or signaling pathways of BDNF have been discussed concerning various neurological and psychiatric conditions [82]. There is also increasing evidence highlighting the significant role of BDNF in visceral pain and visceral hypersensitivity (Figure 2) [99,100,101].

## 3. Role of Probiotic-Derived Metabolites in IBS

**Figure 3:**
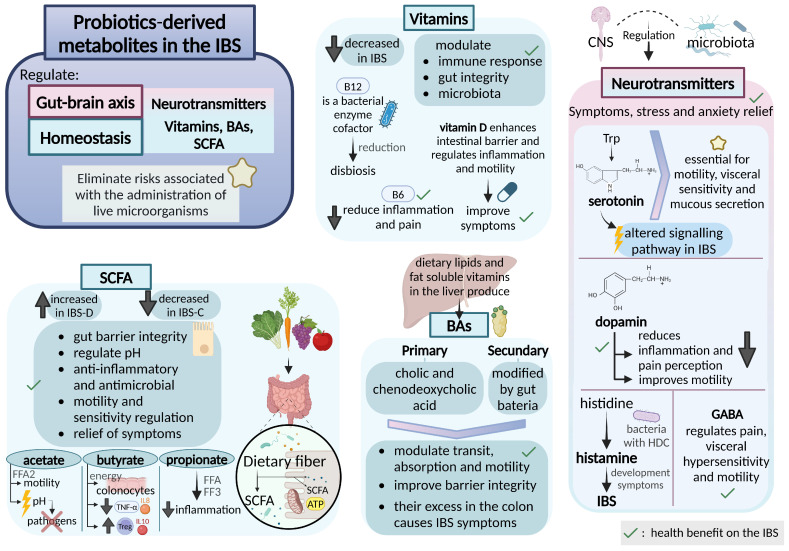
Effects of gut microbiota-derived metabolites, such as vitamins, SCFA, BAs, and neurotransmitters, on the regulation of the gut-brain axis, gut homeostasis, and IBS-associated symptoms. IBS, Irritable Bowel Syndrome; BAs, Bile Acids; SCFA, Short-Chain Fatty Acids [61,102,103,104,105,108,109,110,112,115,117,118,119,120,121,122,123,124,125,126,127,128,129,130,131,132,133,134,135,136,137,138,139,140,141,142,143,144,145,146,147,148,149,150,151,152,153,154,155,156,157,158,159,160,161,162,163].

As commented before, it is widely accepted that probiotics confer a health benefit on the IBS (Figure 3). Interestingly, recent studies indicate that the beneficial effects of probiotics are not solely due to the presence of live bacteria but are also mediated by the bioactive metabolites they produce [102,103,104,105]. These probiotic-derived metabolites, which include SCFAs, bacteriocins, neurotransmitters, and bioactive peptides, play critical roles in maintaining gut homeostasis and may help ameliorate symptoms of IBS. Consequently, growing evidence increasingly highlights the use of probiotic supernatants or postbiotics as a promising therapeutic strategy for managing IBS [106,107]. Postbiotics, active metabolites of probiotics, are essentially the culture media in which beneficial microorganisms, such as *Lactobacillus* and *Bifidobacterium*, have been grown and then removed through filtration, leaving behind a diverse range of metabolites, including SCFAs, proteins, phospholipids, enzymes, peptides, vitamins, bacteriocins, and other bioactive compounds. These metabolites exert various beneficial effects on gut health in IBS without the risks associated with administering live microorganisms [108,109,110]. Specifically, these compounds can influence gut motility, reduce visceral hypersensitivity, and modulate inflammatory responses—factors that are closely linked to IBS symptoms [111,112,113,114]. For instance, SCFAs have been shown to enhance gut barrier integrity and immune function, and BAs are primarily linked to GI malabsorption [109,115,116]. Certain neurotransmitter-related metabolites may help regulate the gut-brain axis, potentially alleviating IBS-related anxiety and discomfort [115]. Recent research suggests that targeting these metabolites offers promising therapeutic potential for managing IBS symptoms and improving patients’ quality of life (Figure 3) [109,110].

### 3.1. Short-Chain Fatty Acids in IBS

Among the most studied probiotic-derived metabolites (PDMs) are SCFAs, including acetate, propionate, and butyrate. SCFAs are produced by the fermentation of dietary fibers, prebiotics, and non-digestible carbohydrates by gut microbiota, particularly by beneficial bacteria and probiotics, such as Bifidobacterium, Lactobacillus, or Faecali-bacterium prausnitzii, among others [117,118,119]. These metabolites have diverse physiological effects that support GI health and may be particularly relevant in the treatment of IBS. Thus, SCFAs act by inducing anti-inflammatory, anti-tumorigenic, and antimicrobial effects, modifying cell proliferation and function, altering chemotaxis and phagocytosis, inducing reactive oxygen species, and modifying gut integrity [164,165,166]. At the small intestine level, SCFAs have been shown to reduce the pH, inhibiting the growth of pathogenic bacteria [120]. Several studies have demonstrated that SCFAs affect gut health directly via enterocytes or by being absorbed into the blood by the gut epithelium, playing a key role in regulating functions related to IBS pathophysiology, such as intestinal barrier integrity, immune modulation, gut motility, and homeostasis [121]. Recent analysis showed how the total concentration of SCFAs was significantly higher in IBS-D patients, showing hyperexcitability and hypermotility, and lower in IBS-C compared to healthy controls [112,121,122,123,124]. These findings can be accounted for in two possible ways: (1) colonic fermentation is enhanced by increased intestinal motility, resulting in higher fecal concentrations of SCFAs; and (2) the reduced transit time delays the absorption of SCFAs, leading to their accumulation [167]. Recent research has found a correlation between probiotic administration and an increase in SCFA production, thereby enhancing the functionality of the intestinal microbiota [60]. This therapeutic potential of SCFAs in IBS is further supported by studies showing that probiotic strains that produce high amounts of SCFAs, such as Bifidobacterium and Lactobacillus species, can significantly improve symptoms of IBS, particularly by alleviating abdominal pain, bloating, and irregular bowel movements [125]. For instance, studies using Bifidobacterium lactis HN019 have shown to improve intestinal dysmotility by modulating the gut-brain-microbiota axis via SCFAs generated by bacterial fermentation [61,62]. These findings underscore the importance of SCFAs as key probiotic-derived metabolites that influence gut health and may have beneficial effects in patients with IBS (Figure 3).

Butyrate, a major SCFA, is a main energy source for colonic epithelial cells and plays a pivotal role in maintaining gut barrier integrity [126,127]. The production of butyrate is associated with the strengthening of tight junctions between enterocytes, which helps prevent increased intestinal permeability and protects against “leaky gut” syndrome, a condition often seen in patients with IBS [128]. Increased intestinal permeability allows for the translocation of harmful pathogens and endotoxins into the bloodstream, contributing to systemic inflammation and exacerbating IBS symptoms. Closely, it has been reported that butyrate also exerts anti-inflammatory effects by downregulating the production of pro-inflammatory cytokines like TNF-α and IL-8 through the inhibition of nuclear factor-kappa B (NF-κB), a key transcription factor involved in inflammatory responses [129]. Additionally, different studies have shown that butyrate reduces intestinal inflammation by inhibiting the production of pro-inflammatory cytokines like IL-12, inducing regulatory T cells, and promoting anti-inflammatory cytokine production like IL-10 [130]. Therefore, in IBS, which often presents with low-grade inflammation, butyrate supplementation has been linked to an improvement in the inflammatory process, which in turn has been associated with enhanced gut barrier function, reduced visceral hypersensitivity, and alleviation of abdominal pain and bloating [167,168]. In this line, recent studies using microencapsulated sodium butyrate have shown to be effective in relieving IBS symptoms by modifying the intestinal microbiota [169]. The main bacteria involved in the production of butyrate are *Clostridium*, *Eubacterium*, *Fusobacterium*, *Butyrivibrio*, *Coprococcus*, *Anaerostipes*, *Subdoligranulum*, *Anaerobutyricum*, *Megasphaera elsdenii*, *Mitsuokella multiacida*, *Roseburia intestinalis*, *Faecalibacterium prausnitzii*, and *Eubacterium hallii* and *rectale*, among others. Additionally, *Actinomycetes*, *Bacteroidetes*, *Proteobacteria*, and *Spirochetes* have also been recognized as potential butyrate-producing bacteria [129,170,171]. The consumption of *Lactobacillus paracasei* CNCM I-1572 has notably shown to modify fecal *Clostridiales* bacteria and butyrate levels in healthy volunteers [172]. Furthermore, this probiotic showed to be capable of significantly reducing the genus *Ruminococcus* and to induce a significant increase in the fecal levels of butyrate in a pilot trial involving 42 patients with IBS (Figure 3) [173].

Acetate is the most abundant SCFA, contributing to approximately 60% of the total SCFAs [174]. Acetate is an agonist for free fatty acid receptor 2 (FFA2, formerly GPR43), which is found in endocrine, immune, and nervous cells along the GI tract [131], and it participates in lipid and carbohydrate metabolic pathways [175]. It has been shown to be involved in pH regulation in the colon, generating an environment conducive to the growth of beneficial bacteria while inhibiting harmful microorganisms [132]. In this regard, in vitro studies have proposed that acetate-producing *Bifidobacterium* can offer protection against bacterial infections [176]. Acetate levels are higher during the first stages of life, as it is the primary metabolite produced by *Bifidobacterium* strains that dominate the infant gut microbiota; strains *Bifidobacterium bifidum*, *Bifidobacterium infantis*, and *Bifidobacterium breve* are the key contributors [177]. The produced acetate supports the proliferation of propionate- and butyrate-producing bacteria, while butyrate promotes the growth of *Bifidobacterium*, resulting in a reciprocal feeding relationship among SCFAs-producing bacteria [178,179]. Fecal SCFAs amounts showed to be different between individuals with IBS, across IBS subtypes, and healthy subjects [180,181,182]. Patients with IBS showing higher levels of acetic and propionic acid presented significantly worse symptoms related to the GI tract, lower quality of life, and stronger negative emotional responses than those with fewer levels or healthy controls [180]. Despite this inconsistency with levels, recent studies have shown that the proportion of fecal acetate with respect to total SCFAs is significantly lower in patients with IBS compared with the healthy controls [167,183]. Moreover, those patients have shown increased numbers of *Veillonella* and *Lactobacillus*, two producers of acetate and propionate [180]. An oral treatment using the probiotic strain *Lactobacillus paracasei* CNCM I-1572 showed to increase acetate levels of patients with IBS as well as significantly reduce *Ruminococcus*, which is normally increased in IBS patients, suggesting an ecological link between these factors (Figure 3) [173].

Propionate has been shown to regulate gut motility and reduce intestinal inflammation by interacting with receptors such as FFA2 (GPR43) and FFA3 (GPR41), being both involved in immune responses and gut motility regulation [133]. These effects may help alleviate symptoms of IBS, particularly in patients with IBS-D slowing down the colonic transit [33,184]. Recent studies have shown that the concentration of fecal propionate in patients with IBS is notably higher compared to healthy controls [167]. Additionally, *Lactobacillus* and *Veillonella*, which are propionate-producing genera, have shown to be more abundant in patients with IBS as they convert lactic acid into propionate [185]. A study testing in mice a new probiotic cocktail containing Lactobacillus and Enterococcus strains obtained from humans has shown to modulate propionate and butyrate production in the gut (Figure 3) [60].

### 3.2. Bile Acids in IBS

BAs are steroid acids synthesized in the liver that are involved in the absorption of dietary lipids and fat-soluble vitamins, influencing the transit time and stool consistency. BAs participate in the endocrine and paracrine functions, regulating lipid and glucose metabolism and modulating temperature and energy homeostasis [134,135]. Among the mechanisms of action of BAs include antimicrobial effects, stimulation of colonic motility, and mucosal permeability [136]. BAs profile is correlated with diet, age, and genes of microbial enzymes and metabolites. It has been studied that the levels of BAs may be associated with visceral pain and colonic transit [137], and recent studies have linked failures in BAs metabolism with the onset of IBS symptoms (Figure 3) [138,186].

Primary BAs, cholic and chenodeoxycholic acid, are de novo synthesized from cholesterol in the liver [138,139,140]. Around 95% of these primary BAs are reabsorbed and recycled via the hepatic circulation [187]. The rest of BAs avoid this step and undergo modification by microorganisms, having as a result secondary BAs with modified structures that may interact with cellular receptors and have important effects on the functionality of metabolites [138]. Disruptions in the BAs reabsorption process would result in the overload of BAs in enteric lumens and lead to digestive disorders [188]. It has been shown that excessive intracolonic BAs are responsible for abdominal discomfort, increased colonic motility, and fluid secretion in patients [141]. Moreover, animal studies have reported that intracolonic exposure to BAs can induce visceral hypersensitivity [189,190]. It follows that the altered BAs metabolism is involved in the development of IBS symptoms, playing gut microbiota a key role in this process [191,192,193]. The conjugate BAs undergo deconjugation by the microbial enzyme bile salt hydrolases, which are mainly present in Gram-positive bacteria *Bacillota* (*Lactobacillus* and *Enterococcus*) and certain Gram-negative bacteria (*Bacteroidota*) [194]. On the other hand, the critical secondary enzymatic step to synthesize secondary BAs from their unconjugated form is performed by anaerobic bacteria (*Clostridium* species) by means of the 7α-dehydratase enzyme [194]. In this sense, recent studies have shown that the use of prebiotics, probiotics, and natural products, as well as diet patterns, might alleviate IBS symptoms by modulating the BAs profile through the microbiota. For example, it has been documented that distinct dietary-BAs patterns affected the gut microbiota and their metabolites. This research demonstrated that a diet rich in animal products, as opposed to a plant-based diet, boosted the concentration of BAs in fecal samples due to higher levels of cholesterol (a precursor to BAs) in animal-based diets [195]. Moreover, they showed that this type of diet enhanced the presence of bile-tolerant microorganisms (*Alistipes*, *Bilophila*, and *Bacteroides*) and reduced the levels of *Bacillota* that metabolize plant-based dietary polysaccharides (*Roseburia*, *Eubacterium rectale*, and *Ruminococcus bromii*) (Figure 3).

On the other hand, it is understood that probiotics engage with BAs in the gut lumen, influencing their metabolism, which in turn results in changes to the pharmacokinetics of numerous pharmacologically active substances [196]. *Lactobacilli* and *Bifidobacteria* subspecies are able to de-conjugate and absorb BAs, as well as perform BA biotransformation, which may detoxify species that are linked with increased intestinal permeability and pain [197]. In this sense, an interventional human study with multi-strain probiotics containing *Bifidobacterium longum* BORI, *Bifidobacterium bifidum* BGN4, *Bifidobacterium lactis* AD011, *Bifidobacterium infantis* IBS007, and *Lactobacillus acidophilus* AD031 has shown that the probiotic administration to patients with IBS leads to a decreased urine level of cholic acid, a primary BAs normally elevated in inflammatory gut diseases [198], indicating a BAs homeostasis stabilization after the probiotic intake [199]. In patients with IBS-D, a mixture of cholestyramine and multi-strain probiotics (with *Lactobacillus rhamnosus*, *Lactobacillus plantarum*, *S. thermophilus*, *Lactobacillus acidophilus*, *Bifidobacterium bifidum*, *Bifidobacterium longum*, *Bifidobacterium infantis*, and *Saccharomyces boulardii*) led to pronounced alterations of BAs metabolism indicators, including composition of serum and fecal BAs as well as increasing of gut bacterial producing bile salt hydrolase-activity (Figure 3) [200].

### 3.3. Neurotransmitters in IBS

#### 3.3.1. Gamma-Aminobutyric Acid (GABA)

GABA is an inhibitory neurotransmitter that has a crucial function in modulating neuronal excitability, and it participates in sleep, anxiety, or motor control, among others. GABA reduces neuronal excitability, helping to balance excitatory processes that could trigger stress, anxiety, or mood disturbances [201]. In the context of IBS, GABA influences both the brain and the GI tract, helping to modulate the communication between them. In addition, GABA contributes to homeostasis and enteric nervous system-related disruptions, like acid secretion, stomach emptying, bowel motility, and pain awareness (Figure 3) [142].

Since stress and emotions are common factors in IBS, GABA plays an important role in how the brain processes and responds to these signals, which may influence GI symptoms [202]. For example, emotional stress can affect gut motility, visceral hypersensitivity, and enteric nervous system functionality, which together can exacerbate IBS symptoms (abdominal pain, bloating, diarrhea, or constipation). Decreased GABA levels can contribute to the development of depression and anxiety disorders in the context of IBS-D by way of mild inflammation [143]. Therefore, boosting GABA activity may help mitigate these symptoms and modulate the excessive response in the GI system, potentially easing abdominal pain and other related symptoms in individuals with IBS [203,204]. Of note, the microbiota could improve the impact of GABA on host cells. In this line, a recent study using in vitro thirteen lactic acid bacterial strains, from *Levilactobacillus brevis*, *Lactiplantibacillus plantarum*, *Lacticaseibacillus paracasei*, *Ligilactobacillus salivarius*, and *Streptococcus thermophiles* species, has shown to modify the gut microbiota composition, increasing the abundance of *Veillonellaceae* and *Bacteroides*, a potential GABA producer related to anti-inflammatory effects [205]. Different reports have demonstrated that long-term administration of *Lactobacillus rhamnosus* JB–1 modified GABA receptor expression in the brain, leading to a reduction in anxiety-like and depressive behaviors [202]. Different strains of GABA producers, such as *Bifidobacterium dentium*, as well as *Lactobacillus plantarum* DM5, have shown capacity to regulate the intestinal hypersensitivity of a rat model [206,207]. Moreover, it has been shown that oral supplementation with strains producing glutamate decarboxylase B, such as *Bifidobacterium dentium* ATCC 27678, results in a reduction in visceral sensitivity in gut and abdominal pain in a rat model (Figure 3) [208].

#### 3.3.2. Dopamine

Dopamine is an essential neurotransmitter for various key functions, including voluntary movements, cognition, reward, satiety, and motivation [209]. Moreover, dopamine controls chronic pain [144] and psychological disorders, and it is able to regulate intestinal inflammation [145]. The dopamine D2 receptor antagonist has been shown to improve GI motility in subjects with IBS [146]. Meanwhile, the dopamine D5 receptor plays a crucial role in increasing the permeability of duodenal epithelial cells and protecting the colonic mucosa [210]. Recent research highlights the important role of gut microbiota in regulating dopamine concentrations through the microbiota-gut-brain axis. Gut microbiota possess enzymes involved in dopamine metabolism, supporting its production and the breakdown of its metabolites [211,212]. Therefore, dysbiosis can affect biosynthesis, secretion, availability, and reuptake of dopamine [213]. As a result, dopamine might contribute to disorders like IBS, where levels of dopamine have shown to be decreased [214]. In this way genera such as *Prevotella*, *Bacteroides*, *Lactobacillus*, *Bifidobacterium*, *Clostridium*, *Enterococcus*, and *Ruminococcus* [215,216,217,218] and several bacterial strains, including *Escherchia coli*, *Klebsiella pneumoniae*, *Pseudomonas aeruginosa*, *Shigella sonnei*, and *Staphylococcus aureus*, have shown to influence dopamine levels by modulating receptors, transporters, and specific targets of the dopaminergic pathway (Figure 3) [219].

Probiotics might influence dopamine levels through the microbiota-gut-brain axis, helping to alleviate both the gut and psychological symptoms of IBS. By improving the balance of gut bacteria, probiotics could potentially restore the function of dopamine in both the peripheral and central nervous systems, leading to improved gut motility, reduced inflammation, and better overall mood regulation. *Lactobacillus* and *Bifidobacterium* strains can enhance GI motility and reduce bloating, which may be linked to their influence on dopamine regulation. *Lactobacillus rhamnosus* can influence the stress response and emotional behavior through dopamine modulation [202]. *Bifidobacterium infantis* M-63 has been shown to be effective in improving the mental health of subjects with IBS due to the restoration of microbial balance and the gut-brain axis (Figure 3) [220].

#### 3.3.3. Serotonin

The gut-brain axis is a two-way communication system between the gastrointestinal tract and the central nervous system, with serotonin serving as a key neurotransmitter. More than 95% of this serotonin is produced in the gut, where it is involved in essential functions like motility, visceral sensing, and mucosal secretion [147]. Therefore, disruptions in serotonergic signaling [221] pathways might be involved in GI disorders, such as IBS. In fact, an increased abundance of serotonin has been reported in the blood of patients with IBS compared with healthy subjects [222]. Because serotonin is mainly produced in the gut, it is not unexpected that its microbiota may play an important role in its availability. In this way, members of the order *Clostridiales* (particularly families *Ruminococcaceae* and family *Lachnospiraceae*) have been involved in stimulating the biosynthesis and release of serotonin and modulating GI motility [148,149]. A recent clinical trial using *Lactobacillus acidophilus* LA-5 and *Lactobacillus paracasei* L. CASEI-01 showed antidepressant properties, as well as alleviating GI symptoms and improving the quality of life of patients with IBS. Its efficacy is suggested by a regulatory mechanism of serotonin involved in restoring gut microbiota [223]. *Bifidobacterium lactis* HN019 has been shown to reduce intestinal transit time and increase bowel movement frequency by modulating the gut–brain–microbiota axis via the serotonin signaling pathway (Figure 3) [61].

#### 3.3.4. Tryptophan

Recent research has concentrated on Trp, the precursor of serotonin, and the function of bacteria in modulating its metabolism. Trp is an essential amino acid that acts as a precursor to serotonin but can also be metabolized via the kynurenine pathway, resulting in the production of other neuroactive compounds [150]. Variation of Trp metabolism has been shown to influence mood and cognition within the central nervous system as well as secretion, motility, and perception in the enteric nervous system [224]. The gut microbiota has shown to be able to metabolize Trp that, in combination with host GI metabolism, are key factors in the systemic availability of Trp, as well as indoles, kynurenine, and serotonin [148,225]. This study remarked on the impact of the gut microbiota on the fate and metabolism of Trp. Importantly, the enzymes responsible for the initial conversion of L-tryptophan to L-kynurenine, indoleamine-2,3-dioxygenase (IDO), and trypto-phan-2,3-dioxygenase may also be regulated directly or indirectly by the gut microbiome [226]. Importantly, IDO activity has recently been shown to be elevated in patients with IBS [227]. In this way, differences in microbial contents have been demonstrated with respect to functional connectivity of brain regions and GI sensorimotor function, implying shifts in the dynamics within the brain-gut-microbiome axis normally shown in patients with IBS (Figure 3) [228].

Probiotics, such as microorganisms from the genera *Lactobacillus* and *Bifidobacterium*, are known to have positive effects on Trp metabolism [229] as well as directly transform Trp into serotonin [230]. Additionally, certain probiotic strains of *Lactobacillus*, like *Lactobacillus casei* 327, may indirectly enhance colonic serotonin production [231]. Consistent with elevated serum serotonin levels, oral administration of *Lactobacillus johnsonii* cell-free supernatant leads to a reduction in kynurenine levels in the bloodstream, along with decreased intestinal IDO activity in rats [232].

In healthy subjects, *Lactobacillus johnsonii* N6.2 intake [233] as well as a multi-strain probiotic including *Bifidobacterium longum* BORI, *Bifidobacterium bifidum* BGN4, *Bifidobacterium lactis* AD011, *Bifidobacterium infantis* IBS007, and *Lactobacillus acidophilus* AD031 tested in patients with IBS-D [199] showed to lead to a reduction in serum kynurenine levels, accompanied by higher Trp levels. Same results were observed after administering Bifidobacterium infantis to rats [234]. These findings suggest that certain probiotic strains may alter host Trp metabolism by inhibiting the kynurenine pathway. On the other hand, Lactobacillus species are reported to be able to break down Trp into indolic compounds [235,236].

#### 3.3.5. Histamine

Histamine is a short-acting endogenous amine that is distributed along the human body, being particularly abundant in the skin, lungs, and GI tract [237]. At the GI tract level, histamine is thought to influence regulation of motility, stimulation of gastric acid secretion, and modification of mucosal ion transport [238,239]. Multiple clinical and animal studies have discovered that histamine levels in the colon were elevated in patients with IBS [240]. In this sense, recent findings indicate that histamine, derived from histidine, may play a key role in the development and symptoms of IBS [151]. Some histamine receptors in the gut are involved in mediating sensorineural signaling, immune responses, and pain perception, being all of them crucial pathways for gut–brain communication. The evidence suggests that excessive histamine production could be responsible for diarrhea resulting from heightened neuronal secretomotor activity [151]. Another theory proposes that in constipated individuals, histamine disrupts enteric neuron function due to excessive segmental contractile colonic motor activity (Figure 3) [241].

Apart from host cells, histamine can also be produced by some strains of microorganisms, such as *E. coli* and *Morganell morganii*. The bacterial synthesis of histamine is facilitated by the histidine decarboxylase enzyme (HDC), which converts the amino acid histidine into histamine. Many Gram-positive and Gram-negative bacteria possess HDC-encoding genes and are thus capable of producing histamine [152]. Relatedly, preclinical and clinical studies suggest that abdominal pain in some patients with IBS may be promoted by fungus-induced mast cell-derived histamine release which subsequently activates the sensitization of histamine receptor 1 (H1) on sensory afferent neurons and the associated nociceptive transient receptor potential channel V1 (TRPV1) [242,243]. Being TRPV1 a major target for the antinociceptive effect of the probiotic *Lactobacillus reuteri* DSM 17938 [244]. Interestingly, some bacteria have the ability to control histamine production by producing histidine decarboxylase, an enzyme that converts histidine into histamine [240]. Moreover, a recent study conducted with a humanized mouse model using germ-free mice colonized with fecal microbiota from IBS patients identified *Klebsiella aerogenes* as a key histamine producer. *Klebsiella aerogenes* was found to be abundant in the fecal microbiota of IBS patients, particularly those with high urinary histamine levels. Moreover, histamine derived from *Klebsiella* contributed to visceral hyperalgesia in mice colonized with microbiota from these patients through histamine H4 receptor signaling, resulting in the buildup and activation of mast cells in the colon [245].

### 3.4. Vitamins in IBS

It has been widely described that microbiome modulation can occur through direct interactions with dietary elements that influence the microbiome’s composition or metabolic activities, or indirectly by altering gut physiology to create changes in the intestinal environment, which in turn affects the microbiome. Vitamins are potential microbiome modulators via multiple pathways. These compounds, classified as either fat-soluble or water-soluble, serve diverse roles in the body. Fat-soluble vitamins, absorbed and transported similarly to lipids, are vital for cell membranes, while water-soluble vitamins generally act as coenzymes in metabolic processes, transferring chemical groups or electrons. Certain vitamins, such as A, B6, C, and E, exhibit antimicrobial properties that can directly influence the gut microbiome, as evidenced by shifts in fecal bacterial profiles (Figure 3) [246].

Vitamins involved in energy metabolism can support specific bacteria by promoting their growth or enhancing their biological activities. Additionally, vitamins can indirectly influence the microbiome by modulating the immune system or altering infection susceptibility, particularly in the gastrointestinal tract [246]. Moreover, the microbiome itself produces vitamins, contributing to micronutrient availability and stabilizing gut bacterial ecosystems [246]. Thus, vitamins can exert bi-directional effects on the microbiome, both directly and indirectly, without serving as an energy source.

In the digestive system, vitamins play key roles in nutrient absorption, gut movement, regulation of the gut microbiome, and other essential functions. In fact, deficiencies in vitamins can disrupt normal physiological processes, leading to gastrointestinal diseases such as beriberi or scurvy. Different studies have highlighted the role of the vitamin in IBS; however, the relationship between vitamins and IBS symptoms remains uncertain. Thus, it has been reported that habitual diet in patients with IBS was associated with deficiencies in vitamins A, B6, and B12, of which patients with IBS on restrictive diets such as low-FODMAP diet also had additional deficiencies in vitamins B1, B2, B9, and D. Interestingly, scores from the different and validated questionnaires, including IBS-quality of life, IBS- Symptom Severity Score, and IBS- Type of Symptoms, improved after vitamin D supplementation [247]. Despite the different studies on management of IBS, only a small percentage analyzed the role of vitamins. More specifically, the effect of vitamin supplementation in alleviating IBS symptoms apart from that of vitamin D is still rather unknown.

#### 3.4.1. Vitamin B12

Vitamin B12 is an essential cofactor for two enzymes in humans: l-methylmalonyl-CoA mutase in the mitochondria and methionine synthase in the cytoplasm. Vitamin B12 supports DNA synthesis, methylation, and folate metabolism, with deficiencies impairing cell division, erythropoiesis, DNA stability, and neurological function (Figure 3) [153].

Recent research indicates that vitamin B12 could play a significant role in the structure and function of the gut microbiome. Results from in vitro studies indicate that vitamin B12 could increase alpha diversity and shift the composition of the gut microbiome [154]. This vitamin is produced and used by bacteria within the gut microbiome, and it is essential for the activity of several bacterial enzymes [153]. Therefore, vitamin B12 deficiency is normally associated with GI symptoms and may contribute to abdominal pain and other symptoms in patients with IBS, making supplementation an option to improve overall well-being and relieve certain GI symptoms. In this regard, several studies have investigated the impact of probiotic intake on vitamin B12 concentrations [248,249,250]. For instance, a randomized nutritional supplementation trial assessing the effect of consuming *Lactobacillus acidophilus* in children indicated enhanced plasma vitamin B12 levels [251]. A multi-center trial involving 62 individuals examined the influence of a customized diet using or not the VSL#3 probiotic on vitamin B12 levels in older adults. The VSL#3 supplement, containing *Bifidobacterium infantis* DSM 24,737, *Bifidobacterium longum* DSM 24,736, *Bifidobacterium breve* DSM 24,732, *Lactobacillus acidophilus* DSM 24,735, *Lactobacillus delbrückii subsp. bulgaricus* DSM 24,734, *Lactobacillus paracasei* DSM 24,733, *Lactobacillus plantarum* DSM 24,730, and *Streptococcus thermophilus* DSM 24,731, showed to improve plasma vitamin B12 levels. Interestingly, subgroup analysis revealed that participants with low-grade inflammation experienced an increase in bifidobacteria levels following VSL#3 treatment [248].

#### 3.4.2. Vitamin B6

Vitamin B6 has been related to inflammatory processes and the development of IBS. Recent studies have shown a significant inverse association between consumption of B6 and the magnitude of IBS symptoms [252], such as inflammation, fatigue, and extraintestinal symptoms, among others [155]. There are different explanations for the observed results. On its part, pyridoxal phosphate 6-azophenyl-2,4-disulfonic acid is a derivative of vitamin B6 and a P2X receptor antagonist, being these antagonists involved in intestinal motility and abdominal discomfort and visceral sensitivity in patients with IBS [156]. On the other hand, low levels of vitamin B6 have been associated with inflammation and proinflammatory cytokines such as IL-6 observed in subjects with IBS [253]. Moreover, pyridoxal 5-phosphate, which is the active form of vitamin B6, participates as a cofactor in cellular metabolism of amino acids, BAs, and lipids [254], with low levels of pyridoxal 5-phosphate associated with a higher IBS symptom score [255]. Different bacterial strains, *Bacteroides fragilis* and *Prevotella copri* (Bacteroidetes), *Bifidobacterium longum* and *Collinsella aerofaciens* (Actinobacteria), and *Helicobacter pylori* (Proteobacteria), have been shown to produce vitamin B6 in the gut (Figure 3) [256,257].

#### 3.4.3. Vitamin D

Vitamin D deficiency is a common characteristic of IBS, with up to 82% of patients affected, and is strongly linked to the onset, progression, and complications of the disorder [258]. This deficiency plays a pivotal role in intestinal barrier integrity, which is often compromised in IBS, as evidenced by disruptions in tight junction proteins [259]. Vitamin D has been shown to preserve barrier integrity by preventing epithelial cell apoptosis and modulating these proteins [157,158,159]. It also exhibits anti-inflammatory properties by inhibiting T helper 1 and 17 cells [260] and downregulating the IL-23 receptor pathway in innate lymphoid cells [261]. Despite its known biological functions, the relationship between vitamin D levels and IBS symptoms remains complex. Reduced serum vitamin D levels in IBS patients have been associated with increased proinflammatory cytokines like TNF-α and IFN-γ, which weaken mucosal barrier function, increasing permeability [259,262,263]. Interestingly, IBS patients show elevated expression of the vitamin D receptor (VDR) in the duodenum, highlighting VDRs role in intestinal barrier function, immune modulation, and the activation of NFκB pathways [264]. Additionally, 1α,25-dihydroxyvitamin D3, the active form of vitamin D, interacts directly with the gut microbiota and mitigates the effects of lipopolysaccharides and TNF-α. However, clinical studies have shown conflicting results regarding the benefits of vitamin D supplementation for IBS. While some trials report significant improvements in symptoms, quality of life, and psychological comorbidities like anxiety and depression [160,161,162,163], others have found no effect on IBS symptomology or overall quality of life. The proposed mechanisms for the observed effects include vitamin D’s ability to reduce low-grade intestinal inflammation, provide mental health benefits, and modulate the gut microbiome. In fact, a recent prospective study has shown that a food supplement combining the three probiotic strains (*Lactobacillus plantarum* CC 7484 and CC 7485 and *Pediococcus acidilactici* CC 7483) and vitamin D in patients with IBS significantly benefited in terms of anxiety, depression, and quality of life [265]. Additionally, several clinical trials have reported that oral Lactobacillus probiotic strains resulted in significantly increased serum vitamin D3 levels [266]. Studies have demonstrated that the probable mechanism behind this is the enhanced production of lactic acid by the probiotic bacteria, which subsequently elevates the enzyme responsible for the absorption and synthesis of vitamin D (Figure 3) [267].

## 4. Conclusions

IBS is a prevalent condition for which no definitive cure is currently available. In recent years, substantial evidence has emerged suggesting a potential link between changes in the gut microbiota and the development of IBS. This has led many experts to advocate for the use of probiotics and their metabolites to manage the various clinical issues related to IBS. While the available evidence is promising, it is still unclear which specific probiotic and/or their metabolites, as well as their combination, are most effective. Additionally, it is uncertain whether probiotic/metabolite treatment should differ depending on the IBS subtype. More research is needed before probiotics and their metabolites can be considered a reliable treatment for IBS. Future studies should be designed with rigorous methodological standards, minimizing bias, and should include enough patients and controls to provide robust evidence. Moreover, long-term follow-up is essential to ensure that the adjuvant treatment is both effective and safe over extended periods.

## Abbreviations

BAs	Bile Acids
BDNF	Brain-Derived Neurotrophic Factor
Foxp3+	Forkhead Box P3
FFA2	Free Fatty Acid Receptor 2
FODMAP	Fermentable Oligosaccharides, Disaccharides, Monosaccharides And Polyols
IBS	Irritable Bowel Syndrome
IBS-C	Irritable Bowel Syndrome Predominant Constipation
IBS-D	Irritable Bowel Syndrome Predominant Diarrhea
IBS-M	Irritable Bowel Syndrome Mixed Bowel Habits
IDO	indoleamine-2,3-dioxygenase
GABA	Gamma-Aminobutyric Acid
GI	Gastrointestinal
GPR43	G-Protein Coupled Receptor 43
GRID2IP	Glutamate Receptor, Ionotropic, Delta 2 Interacting Protein
HDC	Histidine Decarboxylase Enzyme
H1	Histamine Receptor 1
KDELR2	Endoplasmic Reticulum Protein Retention Receptor 2
NF-κB	Factor-Kappa B
PAME	Palmitic Acid Methyl Ester
PDMs	Probiotic-Derived Metabolites
SCFAs	Short-Chain Fatty Acids
TNFSF	Tumor Necrosis Factor Superfamily
Trp	Tryptophan
TRPV1	Transient Receptor Potential Channel V1
VDR	Vitamin D Receptor
